# Remission of lichen amyloidosis achieved with upadacitinib: A case report

**DOI:** 10.1016/j.jdcr.2025.06.061

**Published:** 2025-08-22

**Authors:** Rachel L. Ziebart, Jason C. Sluzevich

**Affiliations:** a Mayo Clinic Alix School of Medicine, Rochester, Minnesota; b Department of Dermatology, Mayo Clinic, Jacksonville, Florida

**Keywords:** lichen amyloidosis, PLCA, primary localized cutaneous amyloidosis, upadacitinib

## Introduction

Lichen amyloidosis (LA) is a form of primary localized cutaneous amyloidosis that primarily affects the lower extremities. It is associated with intractable pruritus and deposition of keratin derived amyloid in the skin that occurs without systemic involvement.^1^ Treatment for LA is challenging, and successful outcomes are primarily anecdotal in nature.^2^ Several case reports detail patients who experienced improvement on dupilumab^3^, ^4^, ^5^ as well as a single report of a patient who responded to upadacitinib,^6^ suggesting biologics may offer therapeutic potential for patients with LA. Herein, we present a patient with treatment refractory LA who had a minimal response to dupilumab, but achieved rapid remission on upadacitinib with early and significant improvement seen after 1 month of therapy.

## Case report

**Fig 1:**
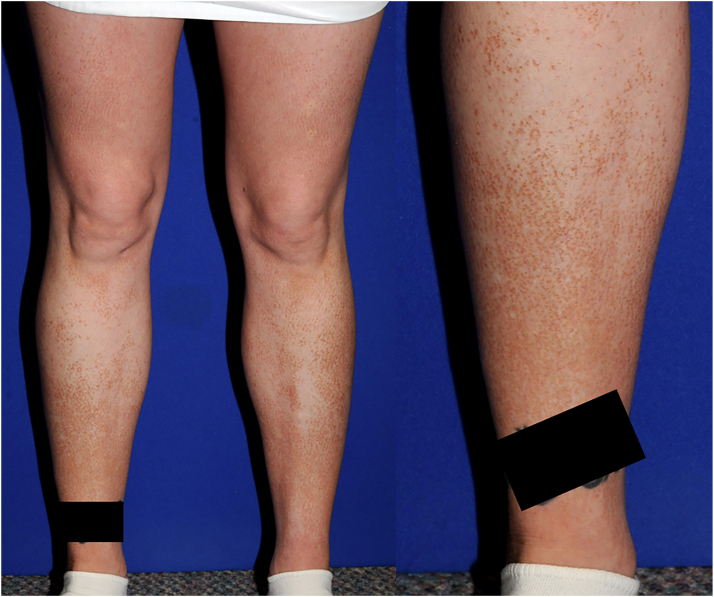
Clinical photos showing lichen amyloidosis on the lower extremities on a female patient in her late 20s. A *black box* covers a tattoo on the patient’s right ankle for protection of patient privacy and artist copyright.

A 28-year-old woman presented with a chronic pruritic eruption of over 10 years duration involving the bilateral lower extremities. Examination of the lower extremities was notable for monomorphic 1 to 2 mm brown to brawny papules that coalesced into cobblestoned plaques, extending from the ankles to the upper midthighs with areas of sparing, as shown in Figure 1.

The eruption was refractory to oral and topical corticosteroids, colchicine, and cryotherapy and symptoms of pruritus failed to improve with first- and second-generation antihistamines. After noticing a small amount of relief after use of the tanning bed at a local salon, she began tanning 3 times per week, which she continued for many years.

Diagnostic evaluation included a chest x-ray, serum protein electrophoresis, serum tryptase, IgE, hepatic function panel, thyroid function studies, and complete blood counts, all of which were unremarkable. A skin punch biopsy from the right side of the lower extremity showed eosinophilic deposits filling the dermal papilla positive for Congo Red and periodic acid–Schiff diastase, and patient was diagnosed with LA. Consideration was given to beginning treatment with an oral retinoid, but due to family planning, the patient requested to treat with a home narrow band UV-B light unit. The home light unit was denied by the patient’s insurance, and she was lost to follow-up for 10 years.

**Fig 2:**
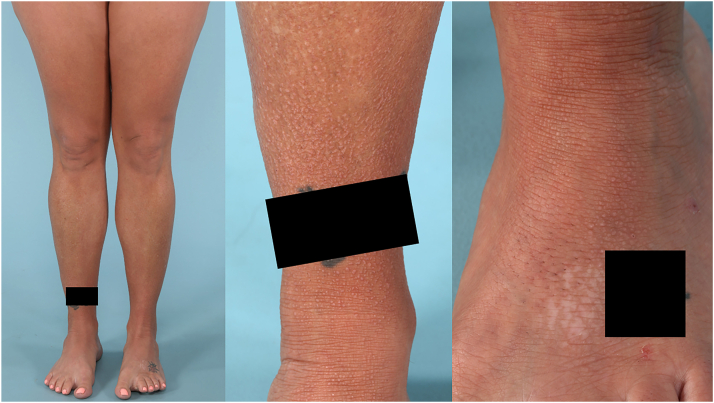
Clinical photos showing gradual worsening of lichen amyloidosis on the lower extremities after 10 years of phototherapy and topical corticosteroids. A *black box* covers a tattoo on the patient’s right ankle for protection of patient privacy and artist copyright.

The patient presented to the clinic again in her late 30s, desiring greater relief than she experienced with thrice weekly tanning and intermittent medium and high potency topical corticosteroids. At this time, skin examination revealed papules that coalesced into hyperpigmented plaques, now involving 40% body surface area with minimal sparing, as seen in Figure 2.

**Fig 3:**
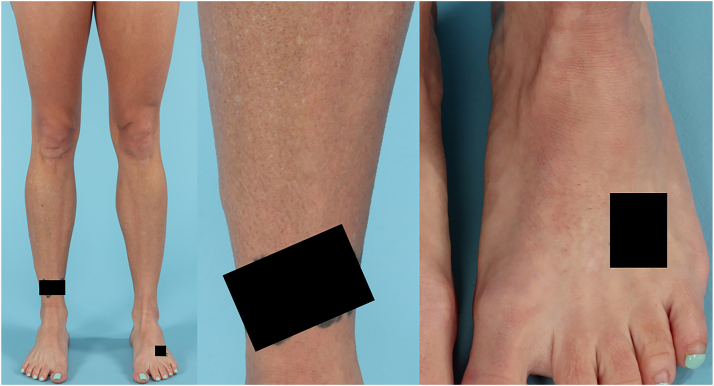
Clinical photos showing near complete resolution of lichen amyloidosis after 6 months of treatment with upadacitinib. A *black box* covers a tattoo on the patient’s right ankle for protection of patient privacy and artist copyright.

Given extent of body surface area involved and severity of pruritus, treatment was initiated with dupilumab therapy. The patient received a loading dose of 600 mg subcutaneous dupilumab, followed by 300 mg every 2 weeks. After 6 months of therapy, the patient had reduction in pruritus but no improvement in the texture or appearance of her skin. Dupilumab was discontinued and the patient began upadacitinib at a dose of 15 mg daily taken by mouth. The patient tolerated upadacitinib well with no adverse effects. After 1 month, she had reduction in both pruritus and the texture of her skin, and after 6 months she had complete resolution of pruritus and near complete resolution of her rash, as shown in Figure 3.

## Discussion

Currently, there is no definitive treatment recommendation for LA available in the literature.^2^ Typical therapies include topical corticosteroids, colchicine and phototherapy,^2^ as well a few case reports of patients who benefited from dupilumab.^3^, ^4^, ^5^ In this case, we describe a patient with chronic progressive LA resistant to corticosteroids, colchicine, and phototherapy who had a minimal response to dupilumab, but near complete resolution with upadacitinib.

Dupilumab is a monoclonal antibody that blocks interleukin (IL)-4 and 13^7^ with therapeutic benefit in a number of skin diseases, particularly those of allergic etiology,^8^ in which IL-4 and IL-13 play a significant role.^7^ By contrast, upadacitinib is a selective Janus kinase inhibitor that blocks phosphorylation of effector proteins in the cytokine signaling pathways, thereby inhibiting downstream inflammation.^9^ Both dupilumab and upadacitinib downregulate IL-31,^10^ a cytokine key in pruritic skin disease. However, evidence from a study in patients with atopic dermatitis suggests upadacitinib may provide greater downregulation of IL-31 than dupilumab.^10^ Although the mechanism of LA is not fully understood, current understanding is that overexpression of IL-31 contributes significantly to the pathophysiology,^1^ which may explain why our patient with dupilumab-refractory LA was able to reach remission on upadacitinib. However, further research in studies with a greater number of patients is needed to confirm the therapeutic effects of upadacitinib for treatment of LA.

LA significantly impacts quality of life, and treatment offers limited success. We report a patient with LA refractory to corticosteroids, colchicine, and phototherapy who had a minimal response to dupilumab, but experienced significant and early improvement on upadacitinib. The patient had reduction in both pruritus and the textural component of her rash after only 1 month of therapy, with near complete resolution achieved by 6 months. This case highlights a new potential treatment option for patients with refractory LA and highlights the importance of the abrogation of the itch-scratch cycle in inducing remission.

## Conflicts of interest

None disclosed.
